# Developing a core outcome set for assessing clinical safety outcomes of cardiovascular diseases in clinical trials of integrated traditional Chinese medicine and Western medicine: study protocol

**DOI:** 10.1186/s13063-022-06166-3

**Published:** 2022-03-28

**Authors:** Ruijin Qiu, Changming Zhong, Siqi Wan, Yao Zhang, Xuxu Wei, Min Li, Jiayuan Hu, Shiqi Chen, Chen Zhao, Zhao Chen, Jing Chen, Hongcai Shang

**Affiliations:** 1 Key Laboratory of Chinese Internal Medicine of Ministry of Education and Beijing, Dongzhimen Hospital, Beijing University of Chinese Medicine, Beijing, China; 2 First Teaching Hospital of Tianjin University of Traditional Chinese Medicine, Tianjin, China; 3 Beijing University of Chinese Medicine Third Affiliated Hospital, Beijing, China; 4 Institute of Basic Research in Clinical Medicine, China Academy of Chinese Medical Sciences, Beijing, China; 5 Baokang Affiliated Hospital of Tianjin University of Traditional Chinese Medicine, Tianjin, China

**Keywords:** Core outcome set, Safety outcomes, Cardiovascular diseases, Integrative medicine, Methodology

## Abstract

**Background:**

Integrative medicine is commonly used in China. Researchers prefer to report efficacy outcomes rather than safety outcomes in clinical trials; thus, evidence regarding safety in integrative medicine is unclear. Developing a core outcome set (COS) for safety outcomes is necessary. In this study, a representative example of the methodology for developing COS to assess safety outcomes of cardiovascular diseases in clinical trials investigating integrated medicine will be developed.

**Methods and analysis:**

Safety information will be extracted from package inserts and through systematic reviews of treatments for cardiovascular diseases (including angina pectoris, myocardial infarction, heart failure, arrhythmia, and hypertension) to develop an extensive list of safety outcomes, which will then be categorized according to whether subjective or objective outcomes. Questionnaires for clinician-reported safety outcomes and patient-reported safety outcomes will be developed. Two rounds of the Delphi survey will then be conducted for different stakeholders (traditional Chinese medicine clinicians and researchers in cardiovascular diseases, Western medicine clinicians and researchers in cardiovascular diseases, integrated medicine clinicians and researchers of cardiovascular diseases, pharmacologists, methodologists of evidence-based medicine, and patients). After round 2 of the Delphi analysis, a face-to-face consensus meeting will be held to determine the final COS for assessing safety outcomes in cardiovascular diseases.

**Discussion:**

A COS for safety outcomes in cardiovascular diseases may improve the consistency of reporting results and will help identify potential bias of selective reporting in the future.

**Trial registration:**

This study was registered in the Core Outcome Measures in Effectiveness Trials database as study 1564.

**Supplementary Information:**

The online version contains supplementary material available at 10.1186/s13063-022-06166-3.

## Background

Safety outcomes are important to help healthcare professionals identify harms of interventions. The Consolidated Standards of Reporting Trials (CONSORT) group has provided recommendations on the appropriate reporting of harms in randomized controlled trials (RCT) [1]. However, researchers have not placed specific attention on reporting adverse outcomes from clinical trials or systematic reviews [2–4], which results in clinical trials failing to provide sufficient safety evidence for future clinical trials [5].

After the extension of the CONSORT statement was introduced into China, the quality of adverse events/reactions reporting was not improved, especially in clinical trials involving traditional Chinese medicine (TCM) [6, 7]. A study showed that 72.1% of the package insert available for TCMs do not report adverse reactions, which significantly interferes with the guidance of clinical practice [8]. In China, 71.2% of patients prefer treatment with integrative medicine (integrated TCM and Western medicine) [9]. Several large systematic reviews of clinical trials for TCM have shown that most interventions involve integrative medicine. However, 67.6% of the package insert for TCM do not report drug interactions [8]; thus, it is always difficult to determine whether the adverse events/reactions are due to TCM or to drug interactions when integrative medicine is used. In the past, when serious adverse events/reactions occurred, some Chinese Patent Medicines were recalled or their distribution was banned by the National Medical Products Administration of China because it was difficult to provide safety evidence supporting TCM when combined in integrated medicine regimens. Although studies showed that Chinese herbal medicines can reduce the toxicity of chemotherapy in patients with cancer, no high quality of evidence is currently available [10].

In addition, China National Adverse Reaction Monitoring Annual Report (2015) showed that more than 50% of serious adverse reactions are due to integrative medicine, but it is difficult to determine the adverse reactions that are caused by TCM themselves, or by new chemical reactions that occur when different medicines are combined, or when the TCM and the associated Western medicine have the same or similar composition, which results in overdose or superposition of effects. Meanwhile, clinical safety outcomes, such as routine blood and urine tests, liver function, kidney function, blood pressure, and electrocardiogram findings, have been reported inconsistency in similar clinical trials; thus, it is difficult to compare or merge clinical safety outcomes for TCM in systematic reviews and meta-analyses.

As a consequence of the aforementioned concerns, it is necessary to develop a core outcome set (COS) for safety data [11]. A COS is an agreed minimum set of outcomes that should be reported or measured in all clinical trials in specific areas of health care, which should include benefits and harms [12, 13]. However, researchers do not currently include safety outcomes in completed COS.

The characteristics of a COS that includes adverse events/reactions are as follows:

1. The scope of the COS is a specific disease with specific interventions [14, 15].
2. No specific safety outcomes are reported, but the adverse events/reactions are defined [16].
3. The COS for safety assessment includes patient-reported symptoms [17].

In this study, we will develop a COS for the assessment of clinical safety outcomes for cardiovascular diseases in clinical trials involving integrated traditional Chinese and Western medicines. We hope this work will contribute to fill the knowledge gaps in methodological research in developing COS.

### Rationale for developing COS for assessing safety outcomes in clinical trials of integrated medicine

Safety outcomes are based on diseases and interventions. In this study, we will develop a COS specific for safety outcomes in cardiovascular diseases, the most common cause of mortality in the world. Cardiovascular diseases are a group of disorders of the heart and blood vessels and include coronary heart disease, cerebrovascular disease, rheumatic heart disease, and other conditions. We included diseases with similar therapies, such as angina pectoris, myocardial infarction, heart failure, arrhythmia, and hypertension.

Interventions including Western and Chinese herbal medications will be extracted from the National Medical Insurance Catalog and the National Essential Medicines Catalog of China, which are commonly used in clinical practice and in clinical trials in China.

Updated package inserts for medicine products are always delayed, and the drug interactions of TCM and Western medicines are unclear. Thus, a systematic review of safety outcomes, including adverse events/effects for TCM and Western medications in cardiovascular diseases, will be conducted to fill the current knowledge gaps.

An extensive list of safety outcomes will be constructed and divided into 3 types:

1. Safety outcomes that are reported in both TCM and Western medicines.
2. Safety outcomes that are reported in TCM.
3. Safety outcomes that are reported in Western medicines.

All safety outcomes will then be classified as objective outcomes (clinician-reported safety outcomes) and subjective outcomes (patient-reported safety outcomes), and questionnaires with specific terminology and using plain language will be developed. All patients will participate in two rounds of the Delphi survey. The final COS will be developed in a face-to-face consensus meeting.

### Scope of the COS

The objective of the study will be to identify safety outcomes to be reported by clinicians and patients specific to cardiovascular diseases, that is, the “what to measure” criteria. This protocol followed the Core Outcome Set Standards for Protocol Items (the COS-STAP Statement) [18].

The scope of the COS for assessing safety outcomes will be as follows:

1. Health status: cardiovascular diseases (including angina pectoris, myocardial infarction, heart failure, arrhythmia, and hypertension)
2. Population: patients with cardiovascular diseases (age: 18–80)
3. Interventions: TCM and Western medicine
4. Context of use: clinical trials, including RCT, observational studies, and case reports or case series

### Registration

The study was registered in the Core Outcome Measures in Effectiveness Trials (COMET) database as study 1564 (available at: http://www.comet-initiative.org/Studies/Details/1564).

## Methods and analysis

### Steering Committee

We will form a national Steering Committee to support this study. The Steering Committee will include six experts and a patient diagnosed with a cardiovascular disease at least 1 year prior. The experts to be included are as follows:

1. A TCM clinician in cardiology, who has a Medical Doctor Degree and more than 5 years’ work experience in a tertiary hospital.
2. A Western medicine clinician in cardiology, who has a medical doctor degree and more than 5 years’ work experience in a tertiary hospital.
3. An integrated medicine clinician in cardiology, who has a medical doctor degree and more than 5 years of clinical experience in a tertiary hospital.
4. A clinical researcher who has a medical doctor degree and research experience in cardiology for at least 5 years.
5. A pharmacologist with a master’s degree and more than 5 years of practice in a tertiary hospital.
6. A methodologist with a medical doctor degree and at least 5 years of work experience.

The Steering Committee will review and confirm the study protocol, settle discussions in case of disagreement among researchers, and review and confirm questionnaires, facilitate the Delphi survey, and will ultimately develop the final COS in the consensus meeting.

### Patient and public involvement

Patients were invited to join the Steering Committee. Patients with cardiovascular diseases will be recruited to participate in two rounds of the Delphi survey and in a face-to-face consensus meeting.

### Design

This COS was developed in three phases.

In phase 1, TCM and Western medicines for cardiovascular diseases will be identified.

In phase 2, an extensive list of safety outcomes for cardiovascular diseases will be established using package inserts of medicine products and systematic reviews from the literature.

In phase 3, we will conduct two rounds of the Delphi survey with different stakeholders.

In phase 4, a consensus meeting will be conducted to determine the safety outcomes that should be measured in the COS.

**Fig. 1:**
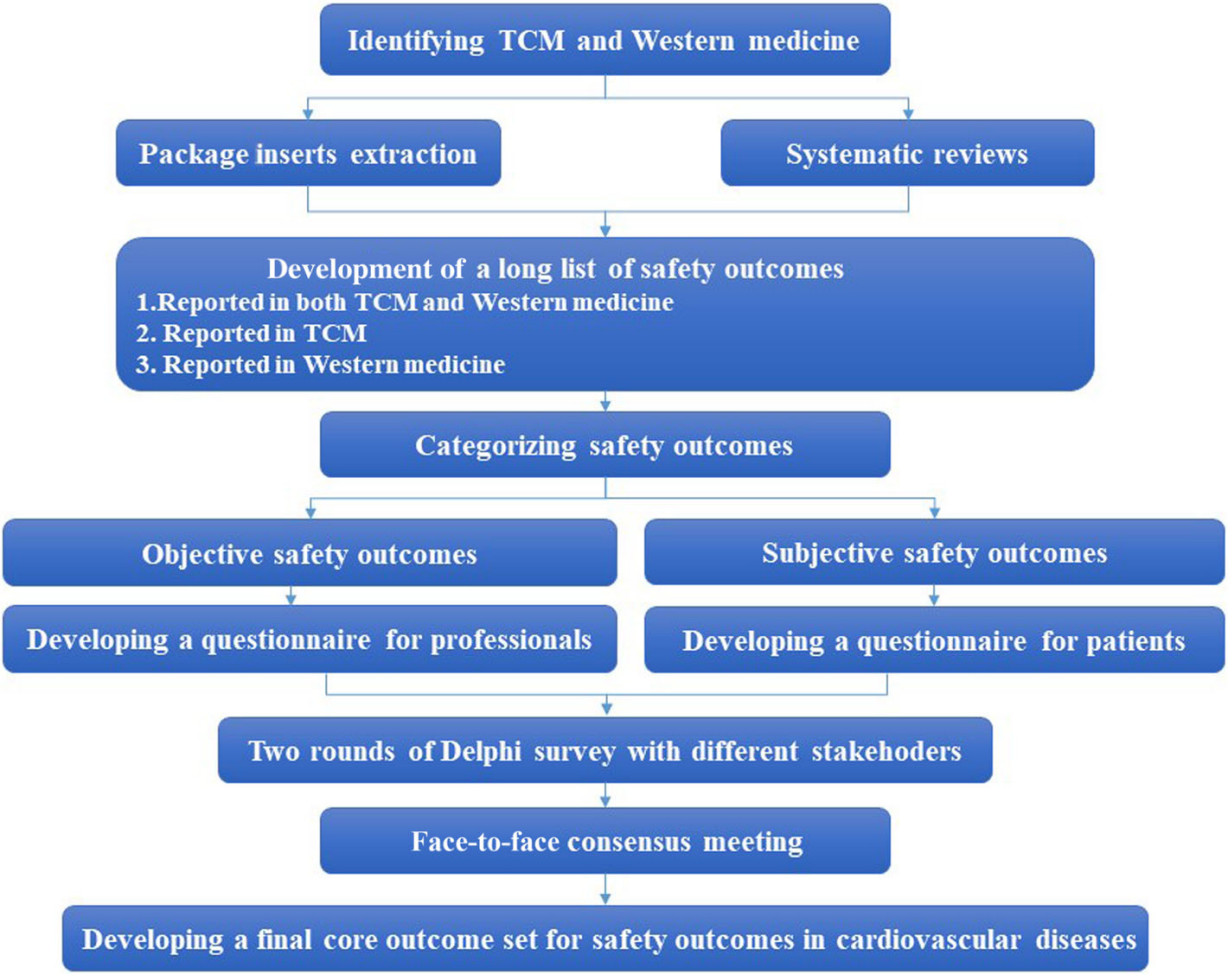
The flowchart of the core outcome set for safety outcomes of cardiovascular diseases. Abbreviations: TCM, traditional Chinese medicine

The flowchart of this study is shown in Fig. 1.

#### Phase 1: Identification of TCM and Western medicines for cardiovascular diseases

A researcher (C Zhong) will identify the Chinese herbal medicines and Western medicines, such as beta blockers, calcium channel blockers, and angiotensin II antagonists, used to treat cardiovascular diseases (including angina pectoris, myocardial infarction, heart failure, arrhythmia, and hypertension) from the National Medical Insurance Catalog and the National Essential Medicines Catalog of China independently. The second researcher (RQ) will review the list of medications.

The methods used for the extraction of the information relative to the interventions are as follows:

1. Information relative to Chinese herbal medications with the effects of promoting blood circulation and removing blood stasis, removing blood stasis and dredging blood, activating Qi and blood, nourishing Qi and blood, calming the liver and extinguishing wind, nourishing the liver and kidney, and nourishing Qi and Yin will be extracted.
2. Western medications, such as beta blockers, calcium channel blockers, angiotensin II antagonists, angiotensin-converting enzyme inhibitors, antithrombotic medications, anti-adrenergic medications, diuretics, antiarrhythmic medications, vasodilators, cardiac glycosides, and antihypertensive medications, will be extracted.
3. Package inserts for each medication will be checked to determine whether the indications included angina pectoris, myocardial infarction, heart failure, arrhythmia, and hypertension. If this information is absent, the medication will be excluded.
4. Medications with the same components in different dosage forms will be extracted as different medications.

#### Phase 2: Development of the list of safety outcomes for cardiovascular diseases

An extensive list of safety outcomes will be developed for cardiovascular diseases: package inserts will be screened and the relevant information will be extracted from systematic reviews relative to TCM and Western medicine in cardiovascular diseases.

##### Step 1: Package inserts extraction

Two researchers (C Zhong and RQ) will extract safety outcomes from package inserts independently. The extraction information will include the names of Chinese herbal medications and Western medications, indications, adverse reactions, contraindications, and notes. The data will be cross-checked and any disagreement will be discussed to achieve a consensus.

##### Step 2: Systematic reviews

Search strategy

Six databases will be searched. English databases will include PubMed, EMBASE, and the Cochrane Library. Chinese databases will include the China National Knowledge Infrastructure (CNKI), Wanfang Data, and China BioMedical Literature Service System (SinoMed). The retrieval period will range from 1 January 2015 to 1 January 2021. The search terms will include diseases, outcomes, and study types, as shown in Table 1. The diseases search included MeSH terms and free words.

**Table 1:** Search terms for systematic reviews of TCM and Western medicine in cardiovascular diseases

Domains	Search terms
Diseases	Angina pectoris; myocardial infarction; heart failure; essential hypertension, arrhythmia; atrial fibrillation; ventricular premature beats; atrial premature beats; tachycardia; bradycardia
Outcomes	Adverse drug reactions; adverse effect; side effect, anaphylaxis; allergic reaction; safety; toxicity
Study types	Clinical trial; observational study, case report, case series, real world study, real world research

Inclusion criteria and exclusion criteria

The inclusion and exclusion criteria for the studies are inserted in Table 2.

**Table 2:** Inclusion and exclusion criteria of systematic reviews for assessing safety outcomes in clinical trials of cardiovascular diseases

Inclusion criteria	Exclusion criteria
Adult patients with angina pectoris, myocardial infarction, heart failure, essential hypertension, arrhythmia, atrial fibrillation, ventricular premature beats, atrial premature beats, or tachycardia, bradycardia	No adverse events/reactions can be extracted
Interventions include Chinese herbal medicines and Western medicines included in the National Medical Insurance Catalog or the National Essential Medicines Catalog for cardiovascular diseases	No causality assessment of adverse events/reactions
Adverse events/reactions should be reported	Full-text cannot be obtained
Randomized controlled trials, observational studies, case reports, and case series	Interventions are operations or other types of non-pharmacological therapies
Literatures published in Chinese or English	

Data extraction

The following data will be extracted: the first author’s name, number of participants, participants’ characteristics, interventions, comparisons, course of treatment, follow-up time, and adverse events/reactions.

Two researchers will independently extract the information. Any disagreement will be resolved by discussion or by consulting with the third investigator (HS).

Merging safety outcomes

After completing the extraction of data from the package inserts and systematic reviews, two researchers (RQ and SW) will merge the safety outcomes according to the definitions. The English name of the safety outcomes will be translated into Chinese. Composite safety outcomes will be extracted as individual outcomes.

Adverse events/effects classification

After the safety outcomes were merged, they will be grouped into 3 types:

1. Safety outcomes reported in both TCM and Western medicine.
2. Safety outcomes reported in TCM alone.
3. Safety outcomes reported in Western medicine.

All safety outcomes will be classified according to whether it was a subjective outcome or an objective outcome. The classification to be used is as follows [19]:

A. Adverse events/reactions based on laboratory/biomarker tests (requires specific equipment), such as lymphocyte counts
B. Adverse events/reactions that can be observed/measured by trained professionals, such as retinal detachment
C. Primarily subjective adverse events/reactions that cannot be observed, such as nausea and stomachache
D. Primarily subjective adverse events/reactions that can be observed, such as vomiting
E. Primarily observable adverse events/reactions that also have subjective components, such as nail discoloration

Categories A and B are defined as clinician-reported safety outcomes. Categories C, D, and E are defined as patient-reported outcomes. However, patients should determine whether the safety outcomes exist for category E, while the clinicians should assess their severity.

#### Phase 3: Delphi survey for different stakeholders

We will conduct two rounds of the Delphi survey for healthcare professionals and two rounds of Delphi survey for patients.

##### Selection of healthcare professionals

The healthcare professionals will include TCM clinicians and researchers in cardiovascular diseases, Western medicine clinicians and researchers in cardiovascular diseases, integrated medicine clinicians and researchers in cardiovascular diseases, pharmacologists, and methodologists in evidence-based medicine.

The inclusion and exclusion criteria for healthcare professionals in the Delphi survey are as follows:

1. Participants with a bachelor’s degree or above who have more than 1 year of work experience.
2. Clinicians with experience in tertiary hospitals, without restriction by geographical area within China.
3. Researchers participating in the design, recruitment of patients, statistical analysis or management of clinical trials of cardiovascular diseases, or who have published at least one paper in the field (first author, corresponding author, or other co-authors).

Exclusion criteria: None.

Healthcare professionals will be recruited from the Dongzhimen Hospital, Beijing University of Chinese Medicine, the members of the Clinical Research Method of Cardiovascular diseases of Professional Committee of the Chinese Association of Integrative Medicine, and the China Research Institute of the China Information Association for Traditional Chinese Medicine and Pharmacy.

##### Patient selection

The inclusion and exclusion criteria for patients are shown in Table 3.

**Table 3:** The inclusion and exclusion criteria for patient involvement

Inclusion criteria	Exclusion criteria
Patients with angina pectoris, myocardial infarction, heart failure, essential hypertension, arrhythmia, atrial fibrillation, ventricular premature beats, atrial premature beats, tachycardia, or bradycardia	Patients with pre-existing severe liver or kidney function damage when they were diagnosed with cardiovascular diseases
Patients were diagnosed with cardiovascular diseases for at least a month	Female patients who are pregnant or breastfeeding
Patients were treated by interventions included in this study	Patients with malignant tumors, diabetes, depression, anxiety, or other mental illnesses requiring long-term treatment
Patients are 18–80 years old	Patients who are difficult to communicate with others

##### Sampling strategy

No standard sample size calculation methods will be performed for the Delphi survey for the COS development. From 12 to 174 healthcare professionals and 32 to 185 patients will be included from previous studies [12]. We will select 20 healthcare professionals at the start of the study, and an additional 100 healthcare professionals will be recruited. Snowball sampling will be used to expand the sample size.

In this study, we will recruit at least 30 patients with angina pectoris, myocardial infarction, heart failure, essential hypertension, and arrhythmia, respectively. Therefore, a total of 150 patients will participate in round 1 of the Delphi survey.

***Questionnaire for healthcare professionals***

All the safety outcomes will be included in the questionnaire for healthcare professionals. However, in the questionnaire, the clinician-reported safety outcomes will be presented in the first part, the patient-reported safety outcomes will be presented in the final part. In the round 1 of the Delphi survey, the participants’ personal information will be collected. The participants will be asked to score the importance of all of safety outcomes using a 9-point scoring system. A score of “1–3” means that the safety outcome is not sufficiently important to be included in the COS, “4–6” means that the safety outcome is important but not critical to be included in the COS, and “7–9” means that the safety outcome is critical and should be included in the COS [13, 21]. In addition, the participants will also have a chance to choose “unclear” if it is difficult for them to determine the importance of the safety outcomes.

***Questionnaire for patients***

Patient-reported safety outcomes will be included in the questionnaire for patients. We will use easily understandable language to substitute medical terminology in the questionnaire. From our previous study, we found that it was difficult for patients to score the importance of outcomes, because they believed that all outcomes were important to them. In this questionnaire, we will not use a scoring system. Patients will simply vote if the safety outcomes are important to them. The patients’ personal information, such as age, sex, diagnosis, and interventions, will be collected in the questionnaire.

***Round 1 of the Delphi survey for healthcare professionals***

Round 1 of the Delphi survey will be carried out online and will last at least 3 weeks. An outline questionnaire will be sent to healthcare professionals by email or WeChat (Tencent), a free social media platform that is universally used in China. We will invite participants to complete Delphi round 1 within 3 weeks. E-mails or messages will be sent to remind them to complete the questionnaire at the end of the second weekend. To improve the response rate, the participants will receive a reward of approximately 30 dollars after completing and submitting the questionnaire. We will send emails or messages to remind potential participants to complete the Delphi survey at the end of the second weekend. If an insufficient number of participants complete the questionnaire, the survey will be kept open for an additional week.

***Data analysis for round 1 of the Delphi survey for healthcare professionals***

Data analysis for round 1 of the Delphi survey will include the response rate, the frequency of the different responses for each safety outcome for each stakeholder group, the score distribution of each safety outcome from the different stakeholder groups will be summarized, and whether the safety outcome achieved a consensus in the different stakeholder groups.

The consensus definition is as follows and has been used in previous COS study [20]:

Consensus in: ≥70% of the participants scored safety outcomes as 7–9, and < 15% of the participants scored a safety outcome as 1–3, which means that the safety outcome should be included in the COS.

Consensus out: ≤50% of the participants scored safety outcomes as 7–9, which means that the safety outcome should not be included in the COS.

No consensus: Values other than the above.

***Round 2 of the Delphi survey for healthcare professionals***

After analyzing the data for round 1 of the Delphi survey, we will conduct round 2 of the Delphi survey. If a safety outcome achieves to the “consensus out” threshold by all stakeholder groups, it will be removed from round 2 of the Delphi survey. The participants in round 2 of the Delphi survey will have a chance to re-score outcomes according to the results of round 1 of the Delphi survey.

Round 2 of the Delphi survey will last for at least 3 weeks. E-mails or messages will be sent to participants to remind them to complete the questionnaire at the end of the second weekend. If the attrition is more than 20%, the survey response period will be extended longer, or we will invite other professionals who did not participate in round 1 of the Delphi survey to participate in.

***Data analysis for round 2 of the Delphi survey for healthcare professionals***

We will analyze the response rate, the score distribution for each safety outcome from different stakeholder groups and all stakeholders, and whether the safety outcome has achieved a consensus. For participants who complete 2 rounds of the Delphi survey, we will calculate the difference in the score between the two rounds.

Attrition bias will be a problem if there is missing data. The attrition bias will be determined by calculating the average score of each outcome scored by the participants who complete or do not complete two rounds of the Delphi survey. The missing data will not be considered if there is no attrition bias. If there is attrition bias, or the participants do not score all safety outcomes, the missing outcomes will be considered as ‘unclear’.

***Round 1 of the Delphi survey for patients***

An investigator will approach eligible patients at the inpatient ward or outpatient department of the Cardiology unit at the Dongzhimen Hospital, Beijing University of Chinese Medicine. The investigator will explain the study to potential patients, and those who agree to participate in the questionnaire will get separate written information sheets and will sign an informed consent form. Then the patients will obtain a printed questionnaire and will complete it with the investigator’s help. Patients who complete round 1 of the Delphi survey will be asked if they agree to participate in round 2 of the Delphi survey, if so, the investigator will collect the email address or WeChat count of the patients. To provide an incentive for the patients to complete the questionnaire, the patients will be informed that they will receive a reward of approximately 5 dollars after the study is completed.

***Data analysis for Round 1 of the Delphi survey for patients***

The data analysis for round 1 of the Delphi survey will include the frequencies of each safety outcome voted by the patients. If a safety outcome is voted by ≥70% patients, it is defined as “consensus in”; if a safety outcome is voted by ≤50% patients, it is defined as “consensus out”; any other value is defined as “no consensus”.

***Round 2 of the Delphi survey for patients***

After analyzing the data for round 1 of the Delphi survey, we will conduct round 2 of the Delphi survey. If a safety outcome is voted by < 15% patients, it will be removed from round 2 of the Delphi survey. The frequencies of each safety outcome voted by patients will be summarized in the questionnaire. The patients will have a chance to re-vote the safety outcomes according to the results of round 1 of the Delphi survey.

Round 2 of the Delphi survey will be conducted by e-mail or WeChat and will last for at least 3 weeks. Emails or messages will be sent to patients to remind them to complete the questionnaire at the end of the second weekend. If the attrition is more than 20%, the deadline will be extended longer, or we will invite other patients to complete it.

***Data analysis for Round 2 of the Delphi survey for patients***

The data analysis for round 1 of the Delphi survey will include the response rate and the frequency of each safety outcome that is voted by patients. The attrition bias will be determined by calculating the frequency of each outcome voted by the participants who complete or do not complete the two rounds of the Delphi survey. The change in the vote between the two rounds will be analyzed.

#### Phases 4: Consensus meeting

##### Stakeholder selection

We will hold a face-to-face consensus meeting after completing the Delphi survey. A total of 20 to 25 professionals and a patient will be invited to attend the meeting, regardless of their participation in the Delphi survey.

The inclusion and exclusion criteria for healthcare professionals in the consensus meeting are as follows:

Inclusion criteria:

1. Healthcare professionals with a master’s degree or above.
2. Healthcare professionals with more than 5 years of clinical experience.
3. Clinicians with clinical experience in tertiary hospitals.
4. There will be no restriction with regard to the geographical area of the professional participants.

Exclusion criteria: None

##### Consensus meeting process

The face-to-face consensus meeting will be held in Beijing, China. It will last one day. An investigator will report the results of round 2 of the Delphi survey. Safety outcomes achieving ‘consensus in’ or ‘no consensus’ will be reported in the meeting. The participants will have the opportunity to discuss any safety outcome if they so desire. The aim of the research is to develop a minimum set for safety outcome in cardiovascular diseases that can be reported by all clinical trials. If there are more than 15 safety outcomes that achieve “consensus in” status in round 2 of the Delphi survey, then all of them will be voted by an anonymous method. If 70% or more of participants vote a safety outcome as important, it will be included in the final COS. If 50% or less of the participants vote a safety outcome as important, it will be excluded. Anything else is considered “no consensus”, and then the Steering Committee will discuss and establish the consensus.

Following the consensus meeting, the clinician-reported COS and patient-reported COS for safety outcomes in clinical trials of cardiovascular diseases will be developed.

## Discussion

This is the first COS for safety outcomes in clinical trials investigating cardiovascular diseases. Safety outcomes are due to interventions, which are different from efficacy outcomes. In this study, we will develop preliminary safety outcomes according to the medications listed in the National Medical Insurance Catalog and the National Essential Medicines Catalog, which are commonly used in China. We believe that these can cover interventions in most clinical trials and clinical practice in China.

After the final COS is developed, it will be published in a journal. The results will be reported at different conferences in China. The findings will also be disseminated on the WeChat Official Accounts Platform of Clinical Research Society of China Information Association of Traditional Chinese Medicine. We will also send a copy of the publication to participants in the study to further the dissemination of the COS.

The strengths of the study include the following:

1. The safety outcomes of TCM, Western medicine, and integrative medicine of cardiovascular diseases are considered.
2. Both clinician-reported safety outcomes and patient-reported outcomes are considered.
3. The researcher can use the COS to compare the safety of different medications when clinical trials include safety outcomes that are also included in systematic reviews/meta-analyses. Furthermore, it is easy to determine whether a selective reporting bias exists in clinical trials of cardiovascular diseases in Western medicine, TCM, or integrative medicine.

The weaknesses of the project include:

1. Medications that are not included in the National Medical Insurance Catalog and the National Essential Medicines Catalog of China will be missed.
2. It will be difficult to consider the incidence of adverse events/effects.
3. Patient involvement will derive from a single center.
4. How to report the safety outcomes is not considered in this study.

### Study status

At the time of the revised submission, the systematic reviews have been completed.

## Supplementary Information

**Additional file 1.**

## Data Availability

Not applicable.

## Abbreviations

COMET: Core Outcome Measures in Effectiveness Trials
COS: Core outcome set
COS-STAP: Core Outcome Set Standards for Protocol Items
CONSORT: Consolidated Standards of Reporting Trials
CNKI: China National Knowledge Infrastructure
RCT: Randomized controlled trials
TCM: Traditional Chinese medicine
